# Barriers to and facilitators of accessing HIV services for street-involved youth in Canada and Kenya

**DOI:** 10.1186/s12889-022-14290-7

**Published:** 2022-10-12

**Authors:** Momina Khan, Katie MacEntee, Reuben Kiptui, Amy Van Berkum, Abe Oudshoorn, David O Ayuku, Edith Apondi, Edward Ou Jin Lee, Alex Abramovich, Sue-Ann MacDonald, Paula Braitstein

**Affiliations:** 1 Division of Social and Behavioral Sciences, Dalla Lana School of Public Health, University of Toronto, Toronto, Canada; 2 Division of Epidemiology, Dalla Lana School of Public Health, University of Toronto, 155 College Street, M5T 3M7 Toronto, ON Canada; 3 Institute for Mental Health Policy Research, Centre for Addiction and Mental Health, Toronto, Canada; 4 Academic Model Providing Access to Healthcare (AMPATH), Eldoret, Kenya; 5 Arthur Labatt Family School of Nursing, Western University, London, Canada; 6 Department of Mental Health and Behavioral Sciences, School of Medicine, College of Health Sciences, Moi University, Eldoret, Kenya; 7 Department of Child Health and Paediatrics, Moi Teaching and Referral Hospital, Eldoret, Kenya; 8 École de Travail Social, Université de Montréal, Montréal, Canada; 9 Division of Child and Youth Mental Health, Department of Psychiatry, University of Toronto, Toronto, Canada; 10 Department of Epidemiology and Medical Statistics, School of Public Health, College of Health Sciences, Eldoret, Kenya

**Keywords:** Street youth, Homelessness, HIV, Access to care, Kenya, Canada, Stigma, Poverty

## Abstract

**Introduction:**

UNICEF estimates that there are as many as 100 million street-involved youth (SIY) globally. Marginalized conditions put SIY at higher risk of HIV and adverse outcomes once HIV-positive. The objective of this analysis was to describe barriers and facilitators of accessing HIV prevention, testing, and treatment services as Phase I of an implementation study evaluating the use of peer navigators to increase access to HIV services.

**Methods:**

Semi-structured interviews, focus group discussions (FGD), and theatre testing were conducted with individuals who identify as SIY, health care providers, and community stakeholders living in Canada (Toronto, Montreal, London) and Kenya (Eldoret, Huruma, Kitale). Data were analyzed using a directed content approach, guided by the socio-ecological model (SEM).

**Results:**

Across the six sites were 195 participants: 64 SIY, 42 healthcare providers, and 97 community-based stakeholders. Barriers were identified at the societal (e.g. intersectional stigma and discrimination), public policy (e.g., inadequate access to basic needs, legal documentation, lack of health insurance, and limited community-based funding), institutional (e.g. lack of inclusive education and training, inadequate HIV educational outreach, and restrictive service provision), interpersonal (e.g., ineffective communication from healthcare providers), and intrapersonal levels (e.g. lack of trust and associated fear, low perception for healthcare, and lack of self-esteem). These contributed to limited HIV services utilization among SIY. Conversely, numerous facilitators were also identified at the public policy (e.g. affordable HIV services and treatment), institutional (e.g. available and accessible HIV prevention tools, HIV education and awareness programs, and holistic models of care), interpersonal level (e.g., systems navigation support, peer support, and personal relationships), and intrapersonal levels (e.g. self-efficacy) as positively supporting SIY access to HIV services.

**Conclusion:**

Intersectional stigma was a critical barrier in all sites, and policies and programs that foster welcoming environments for youth from diverse backgrounds and living circumstances may be better able to respond to the HIV service needs of this high risk population. Social support and navigation services were reported to facilitate access to HIV services in all sites.

**Supplementary Information:**

The online version contains supplementary material available at 10.1186/s12889-022-14290-7.

## Introduction

Access to equitable healthcare has been understood and accepted as a human right [1]. Yet, several disparities experienced by some groups persist to inhibit this right from being actualized [2]. Children and youth who are street involved, experiencing homelessness, precariously housed and/or living on the streets are one such group. Globally, UNICEF estimates that there are as many as 100 million street-involved youth (SIY) [3]. Defined as children and adolescents who spend a substantial amount of time on the street, are heavily engaged in the street economy, and may be absolutely or periodically at risk of becoming homeless, SIY are often victims of abuse and exploitation internationally [4, 5]. In Kenya there are as many as 300,000 SIY who are marginalized by human rights violations and often experience poor health outcomes [6, 7]. In Canada, it is estimated that 35,000 youth are homeless each year [8]. Two-Spirit, Lesbian, gay, bisexual, transgender, queer, and questioning (2SLGBTQ+) youth are disproportionately represented among youth experiencing homelessness, making up to 20–40% of the youth homeless population across Canada [9, 10].

While traditional beliefs regarding why youth may become street involved are often centered on youth delinquency, research shows a more complex socio-structural picture with issues such as poverty, family conflict, and abuse as the most common drivers [11]. Multiple marginalized youth live in vulnerable circumstances interacting with street communities where risks create adverse health outcomes [12]. SIY experience high rates of sexual violence and coercive sexual situations, often with limited knowledge and access to safer sexual practices [13–15]. These youth are then at higher risk for outcomes such as unwanted pregnancies [16] and sexually transmitted diseases, including hepatitis C and HIV [13, 17–19]. A Canadian surveillance report showed that the prevalence of HIV in youth aged 15–25 who used injection drugs was 15 times higher than the national average [12, 20]. In Kenya, the national prevalence of HIV among the general youth population is between 1.1 and 3.0%, and is much higher among SIY even compared to other high risk youth populations such as those who are orphaned [19, 21, 22]. Compared to those living in a family-based setting, individuals living on the street have significantly higher rates of death and incident HIV [19]. Notably, HIV is a leading cause of death among SIY in Kenya [23]. If the UNAIDS targets of 95% of persons living with HIV knowing their status, 95% of them receiving antiretroviral treatment (ART), and 95% of them being virally suppressed are to be met, it is imperative that these high risk and marginalized populations be effectively engaged in available HIV services [24].

The Socio-Ecological Model (SEM) describes how health inequity results from an interplay of elements beyond individual characteristics and behaviours. This is especially important among the SIY population because of the context in which they live. As a key framework in health promotion, McLeroy et al. [25] outlined five levels of the SEM that influence healthcare access, including intrapersonal (e.g., knowledge, attitudes), interpersonal (e.g., formal and informal supports), institutional (e.g., rules and regulations for operations), community (e.g., networks), and policy factors (e.g., laws and policies). More recent iterations of this model also consider societal factors (e.g., social and cultural norms) [26].

As a model used to understand the interdependence of cultural, environmental, political, organizational, and biological drivers for behaviour and incidence of disease [27], the SEM has been widely implemented to understand HIV across adult and some youth populations [28]. However, there is limited knowledge on access to HIV services among SIY globally. Research has shown that despite the numerous HIV interventions targeted towards youth, the engagement of youth themselves in the development and implementation of programs is challenging and limited [29–32]. The “one size fits all” approach to existing youth targeted HIV interventions has been highlighted as an important limitation for youth who are highly diverse in their background and needs [33]. Therefore, it is vital to engage youth to design interventions and understand gaps. This study used the SEM to examine the barriers and facilitators to accessing HIV services shared from the perspective of SIY themselves, as well as healthcare providers and community workers in Canada and Kenya.

## Methods

### The peer Navigator Project (PNP) (www.pnpstudy.net)

The PNP is a longitudinal, multi-site, implementation science study designed to test the adaptation and scale-up of Peer Navigators in three cities in Canada (Toronto, Montreal, and London), and three cities/townships in Kenya (Eldoret, Huruma, Kitale) to increase uptake and utilization of HIV services (prevention, testing, treatment). Phase 1 of this study was dedicated to engaging a range of stakeholders in assessing the appropriateness and acceptability of Peer Navigators (PN) to support this population in HIV services, adaptations needed, and the barriers and facilitators associated with accessing HIV services in these locations. The Toronto and Montreal sites are specifically dedicated to engaging SIY aged 16–29 years who self-identify as 2SLGBTQ+, while the other sites are targeting the general population of SIY aged 16–29 years. All sites include high numbers of underserved at-risk SIY who subsequently bear a disproportionate burden of uncontrolled HIV [34, 35].

### Human subjects protections

The study was ethically reviewed and approved by all affiliated institutions. Participation in the study was voluntary, confidential, anonymous and each participant was offered honoraria to financially compensate for their time and transportation related costs (Canada $30 CAD; Kenya 1000KES). All participants provided written informed consent.

### Participants

To attain a broad range of information-rich perspectives, convenience and purposeful sampling were used for recruitment [36]. In Canada, participants were recruited in close collaboration with partnering organizations and their networks, using electronic flyers distributed through electronic mailing lists, in newsletters, websites and at centers that serve SIY. In Kenya, participants were recruited through existing community networks and community mobilization. In this study, participants included those who identified as SIY, community stakeholders, and healthcare providers. To participate in this study, SIY were required to (a) be aged 16–29 years, (b) self-report to have spent the majority of their days and/or nights on the street or with other SIY in a shared shelter for at least the past 30 days, and (c) in Toronto and Montreal sites, identify as 2SLGBTQ+. Community stakeholders and healthcare providers were required to (a) have been working with SIY for at least 3 months, (b) speak English, French, or Swahili (depending on the site), and (c) be aged 18 years and above. Individuals provided written consent prior to participation.

### Sources of data

Data collection was conducted between August 2018 and June 2020 by a diverse group of project team members (Toronto: AA, KM; Montreal: RFM, EOJL, London: AVB; Eldoret and Kitale: RK, DM) in terms of sexual orientation, gender identity, race and ethnicity, country of origin, and educational status. All members of the data collection team had experience working in the area of homelessness and/or with SIY in their respective sites. Individual fieldnotes were collected to support reflexive engagement in the data collection process.

Key informant interviews as well as focus groups and theatre testing were used to gather fulsome qualitative data from SIY, community stakeholders and healthcare providers. Participants chose their preference for key informant interview or focus groups. Key informant interviews followed semi-structured question format and averaged 1 h in duration. They were conducted in person or via Zoom after the outset of the COVID-19 pandemic. The questions enquired into SIY’s healthcare needs and their access to HIV prevention, testing and treatment. Interviews were transcribed verbatim and verified by the interviewers for accuracy. Interviews conducted in French or Swahili were translated to English by professional third-party translators.

In Eldoret, Kitale, London and Toronto, FGD’s were conducted in person and averaged one hour in duration. The semi-structured format asked the same questions as the key informant interviews. Groups were separated according to SIY and community stakeholder status. Healthcare providers only participated in interviews, and none chose to participate in a focus group.

Theatre testing took on average 1.5 h to complete and involved screening short video recordings of actors depicting PNs providing support to SIY to respective audiences of SIY, healthcare providers, and community stakeholders [37]. Distinct videos were created for each site in Canada and the Kenyan sites used the same video to reflect the qualities of the different study sites and to aid with data collection during COVID-19 [38, 39]. A total of 15 theatre testing screenings were conducted, 9 were held in person and 6 screenings in Montreal were over Zoom. Discussion questions followed each scenario to prompt audience discussion. Questions explored what the participants perceived to be the optimal PN characteristics (e.g., ideal age, race, gender identity, and sexual orientation) and responsibilities (e.g., accompanying SIY into examination rooms, SIY recruitment and engagement) as well as the optimal environment for the PN to work in (supervision, debriefing, support). Discussions were recorded and transcribed in preparation for analysis alongside the focus group and interview data.

### Analysis

Analysis began with open coding. To construct the codebook, seven researchers from the Canadian and Kenyan sites read one transcript from the three stakeholder groups to identify dominant nodes. Separate codebooks were created in both countries and were then compiled and synthesized. Each code was defined and approved by the team. Using Nvivo 12, The team piloted the compiled code book using two transcripts (one Canadian and one Kenyan) and minor revisions to code hierarchy and definitions were applied. A team of coders (JFB, MK, RK, HK, KM, AVB) read and coded all the transcripts. The team met regularly to maintain consistency in the coding process. Subsequent rounds of coding resulted in minor changes to the codebook and the generation of theme memos. Coded data went through two rounds of review by the coding team to ensure consistency across sites. Guided by directed content analysis, the SEM was used as framework to identify common themes that act as barriers and facilitators for SIY accessing HIV services [39].

## Results

There were a total of 53 interviews conducted, 11 FGD’s, and 11 theatre testing encounters. From a total of 203 participants, 62 were in Kenya, and 141 were in Canada. Overall, 64 identified as SIY, 42 were healthcare providers, and 97 were community stakeholders. Participant characteristics are detailed in Table 1.

**Table 1:** Participant Demographics

Site Location	Source of Data	Category of Participant			Total Number of Participants
		**SIY**	**HCP**	**CS**	
Toronto	Interviews	7	5	5	17
	Focus groups	0	0	12	12
	Theatre Testing	6	0	10	16
Montreal	Interviews	3	4	7	14
	Focus groups	0	0	8	8
	Theatre Testing	0	5	17	22
London	Interviews	3	5	7	15
	Focus groups	11	0	0	11
	Theatre Testing	10	7	9	26
Eldoret/Huruma	Interviews	0	1	3	4
	Focus groups	0	5	5	10
	Theatre Testing	12	0	0	12
Kitale	Interviews	0	1	2	3
	Focus groups	12	3	6	21
	Theatre Testing	0	6	6	12
**Total**		64	42	97	203

**Fig. 1:**
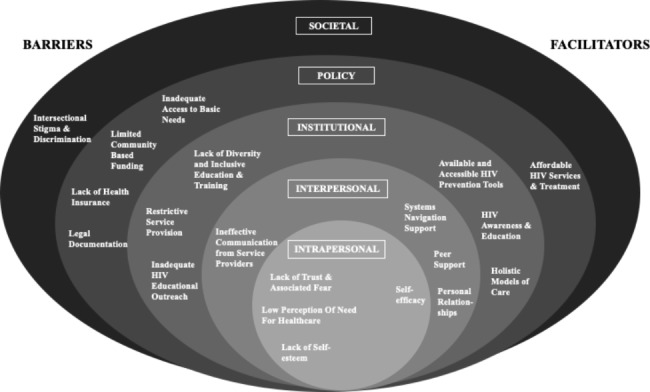
Barriers and Facilitators to HIV Service Access in Canada and Kenya

Themes were proposed inductively from the data while being organized deductively according to the five levels of the SEM: societal, public policy, institutional, interpersonal, and intrapersonal levels (Fig. 1). Here we report findings in two sections, one detailing the barriers and the other detailing the facilitators (Tables 2 and 3), each according to the SEM level. Dominant themes across levels are bolded.

**Table 2:** Common barriers in Canada and Kenya

Level	Theme	Illustrative Quotes
Societal Level	Intersectional Stigma and Discrimination	“[They] feel like they’re targeted, that they don’t get the same care, and that they’re treated as second-class citizens.” (CS, London)
		“For trans youth of colour it’s extremely difficult because there’s so much inherent transphobia in the service provided.” (CS, Montreal)
Public Policy Level	Inadequate Access to Basic Needs	“It becomes a privilege to then think about your medical needs and your mental health needs. It’s more about, ‘Where am I going to be safe? What am I going to do to be warm and to have something over my head, to find a meal?’…I don’t think that people are having the luxury to think about, ‘I’m going to go get my blood work done. I’m going to go get tested,’ you know?” (HCP, Toronto)
	Limited Community-Based Funding	“The one-size-fits-all model doesn’t work but the program didn’t have enough capacity to tailor specifically for that one group.” (CS, Toronto)
		“I’ve had to cut so many programs because we’re in deficit actually…we went from two tokens to one….we had to cut some of our meals or dinners and it’s empty here those nights because they have to find somewhere else to eat and that pulls at my heart strings. It bothers me so much.” (HCP, Toronto)
	Legal Documentation	“STI testing here, we can do without a health card, but anything beyond that, they’re going to need it, and certainly, if they test positive for something like HIV and need treatment, of course they need it.” (HCP, London)
		“[They] feel like that is a huge thing for trans people especially if their IDs have their dead name on it or their previous gender, they don’t want to be misgendered when they go into a clinic.” (CS, Toronto)
	Lack of Health Insurance	“If it’s not covered under OW’s prescription benefits plan, they can’t afford it…. even something like Ontario Trillium benefits is possible, but there’s a large wait for applications to be processed for them to be able to access all covered medications. And if there’s no generic one and they have to pay for it, they just won’t do it.” (CS, London)
Institutional Level	Lack of Diversity and Inclusive Education and Training	“I feel like the harm reduction scene in general is very white, very male dominated and if you’re not from those identities it can kind of feel isolating or people might not feel as welcome to access the services there.” (CS, Toronto)
		“Even within our own team, the people who don’t really know much about PrEP… I think there’s a lot of work that needs to happen around educating people.” (HCP, Toronto)
	Restrictive Service Provision	“The other barrier is…you find there is a queue in the facility or hospital which will take you a lot of time that’s time wastage.” (CS, Eldoret)
		“It’s hard to find a doctor that takes patients…and so I always go to the emergency, but they treat me like I’m just a piece of shit” (SIY, London)
	Inadequate HIV Education Outreach	“STI and HIV testing facilities at some community-based organizations were separated by gender so “someone who might be non-binary might not be sure which drop-in to go to in that case” (CS, Toronto).
Interpersonal Level	Ineffective Communication from Service Providers	“They talk down to us…They don’t let you finish your thoughts, like if you just say two of the 10 symptoms and they go, “Oh that’s this”, I’m like, “You didn’t even hear the other eight”…They don’t really care.” (SIY, London)
		“The doctor sort of explained [HIV], but didn’t get too far into detail, which didn’t help me at all.” (SIY, Toronto)
Intrapersonal Level	Lack of Trust and Associated Fear	“I know that like science and everyone says that like HIV is not a death sentence but it kind of feels like it. It’s a heavy fucking load to hear like, you’ve got HIV so, like they’ll get tested for it and then even just the wait for the results is like am I going to die or am I not going to die? It’s like I think a lot of people are like I already have enough shit that I’m dealing with, I don’t want to hear that… like I really don’t have a house to live in I also don’t want to hear that I’m not doing well health like health wise.” (SIY, Toronto)
		“There is a belief that when you don’t know you are not stressed but once you get tested that is your end so that is believe which may contribute for them not to go for testing.” (CS, Kitale)
	Low Perception of Need for Healthcare	“You can’t blame someone for catching a cold…no one does that, no one is out here blaming people for catching a cold…Healthcare professionals need to understand that people aren’t willingly uneducated, it’s not random. It isn’t surprising that the information never got to those people. Actually, this information is kept from those people, and it’s a responsibility that healthcare workers have. It’s not the individual’s fault that people don’t have this information, and they shouldn’t be expected to go and get it.” (SIY, Montreal)
	Lack of Self-Esteem	“I would have unprotected sex with someone, and I was like ‘that’s what you deserve to live with.’”(SIY, Montreal)
	High risk behaviours	“Someone with really severe mental health and high substance use is going to be unwilling to take their medication, or unable to take their medication. So you can say to that individual hey, you want your ARV today and by and large they’ll say yes, but we may not be able to find that individual, they may be substance using so heavily that today is not a good day for them….I’ve had some instances where someone is so heavily substance using that they may not know where they put their medication, or they may get a month’s worth of medication and it’s like still at the pharmacy” (HCP, London)

**Table 3:** Common facilitators in Canada and Kenya

Level	Theme	Illustrative Quotes
Societal Level	No facilitators were identified on this level by study participants	
Public Policy Level	No additional quotes to report on this level	
Institutional	Available and Accessible HIV Prevention Tools	“I think that’s one reason why our STI clinic is so successful, because it’s not appointment, it’s just drop-in.” (HCP, London)
		“I think a great thing is the anonymity of getting tested and the fact that you don’t need a health card to do the testing…they don’t want to be stigmatized in their community. I think having the anonymous rapid testing, the fact that you don’t need your health card is huge – because many of our young people lose their health card.” (CS, Toronto)
	HIV Awareness and Education	“After a good talk or adherence counseling, they are willing to start their medicine” (CS, Eldoret)
		“A big push for education has been around not sharing a wash or a filter which has been great because the majority of folks who inject, know not to share a needle. But really didn’t necessarily know that…The education piece, at least here, and I’m sure in all of Ontario, has really been pushed, over the last number of months, and I think it’s making a difference.” (HCP, London)
	Holistic Models of Care	“You’re not just dealing with the medical issue” (CS, Montreal)
		“I’ve been in hospitals situations where a social worker has come with me and they sat down and they explain to [healthcare provider], like, “She goes by she, not him, not he. She goes by she/her, her name is Sarah,” and like, they’ve sat down with me the whole time, and made the whole experience a little bit better.” (SIY, Toronto)
Interpersonal Level	Systems Navigation Support	“They just tried to reassure me that even if I was a positive it’s not a death sentence anymore or considered a death sentence. It’s actually something that you can treat and have a full life with now, so it’s – try to calm down” (SIY, London)
		“My first test was I’m gay, I’m having sex with other men, and I want to see how [the healthcare provider’s] reaction was, he was like, oh, so like what does that look like. So, I talk to him and he’s willing to listen to what my experience was, and then I told him about PrEP, and he didn’t actually know about it at the time. Then I went in the next time and he’s like, oh I researched PrEP, and he told me stuff I didn’t even know, I was like, wow so, like he listens.” (SIY, London)
	Peer Support	“Some have seen their peers who are now healthier and are put in support system and they wish to be going to school some are even incur feeding well opportunity of getting other services beyond health has motivated them to go for testing. Also their colleagues have gotten better after receiving treatment.” (CS, Kitale))
		“Peers have a lived experience that carries a lot of weight with everyone. So, I think that’s a strength that they have that clinicians and our service providers may not have and that’s very important.” (HCP, Toronto)
	Personal Relationships	“They want to get families, to continue living and to get healthier so they will want to access that medication……For what I have seen maybe they have fallen sick and they also want to form families so they will want to make sure they are ok, and their partners are okay to enter into a family way” (CS, Eldoret)

### Barriers to HIV services

#### Societal

**Intersectional stigma and discrimination** is a term used to explain the convergence of various identities and the inequities that can result from them [40, 41]. Negative social beliefs and discourses about SIY, their marginalized identities, the high-risk behaviours in which they may engage, and HIV itself presented as dominant barriers to accessing care for SIY across all sites. “They’re dealing with not only with like the stigma of being HIV-positive but the stigma of being queer and/or trans and street-involved” (CS, Montreal). Various stakeholders shared how youth felt hesitant of the medical system because of “being red flagged”, “not being heard, not being taken seriously”, and general “suspicion of the establishment”. “For me, it’s just a challenge even to go get checked out for HIV or anything like that, ‘cause like, I don’t want to be put down or be looked at like I don’t matter, or I don’t care” (SIY, Toronto).

Participants also described healthcare providers stigmatizing youth who disclose engaging in behaviours that put them at greater risk for contracting HIV. A community stakeholder from Montreal described how being honest about sexual health “has always been a barrier” specifically when it comes to “sex work, barebacking, being seropositive, and even prison experience…I always felt like I’d be judged”. Another community stakeholder described stigma related to needle use:

“Lots of folks that are on medication, but if they disclose that they’re doing coke…to the doctor while they’re also being prescribed, the doctors have been known to pull the medication and so it just silenced them” (CS, Toronto)

Stigma and discrimination are often compounded for SIY who are testing for or living with HIV. One youth described how “A lot of places you go to these days make it seem as though taking an HIV test is something wrong” (SIY, Toronto). Several community stakeholders noted how stigma causes youth to avoid getting tested for HIV because “their results would be reported and they would be worried about other people finding out” (CS, Toronto). In Kenya, a community stakeholder from Kitale described how HIV positive SIY are “being called chokora in the street [and] they absorb it [and] own it and for some reason they will not take care of themselves.” (CS, Eldoret).

#### Public policy

Barriers at the policy level included the lack of community and government support resulted in **inadequate access to basic needs** such as stable housing, food, and unemployment, and presented a significant barrier for visiting the doctor, getting tested, and adhering to HIV treatment across all sites in Canada and Kenya. As one SIY described, “Someone who’s in a stable situation can be like, ‘it’s time for me to go for my yearly physical and be okay with making an appointment’, but it’s like if things aren’t stable for [me]… I think I’d rather figure out like what I’m going to eat today or figure out like where I’m going to sleep” (Toronto). A community stakeholder in Kitale explained the need for a stable dietary intake when on HIV treatment: “Another thing also is food, if there is a diet to go with medication, how will they get this medication?” Precarious housing situations and not having a safe place to store belongings played a significant role in limiting the ability of SIY to adhere to ART. “Not having somewhere safe to keep their medication”, “wanting to hide their medication from people”, and “having belongings stolen” were some of the reasons why youth were not able to take their medications in a timely manner.

Additional barriers included **limited community-based funding** wherein budget cuts and policies that restrict funding made it difficult for community-based organizations to reach SIY, connect them to appropriate resources, and educate diverse youth (Table 2). The **lack of health insurance** and the associated costs of HIV services and medications was another dominant barrier as SIY were described as being “afraid to go to hospital because they feel they will be asked for money” (Kitale; Table 2). The need for **legal documentation** to access care presented as a barrier as youth described “sometimes losing their documents.” Youth who identified as 2SLGBTQ + also did not want to show ID that did not have their chosen name and correct gender (Table 2).

#### Institutional

Barriers at the institutional level included a **lack of diversity and inclusive education and training among healthcare providers and other service providers.** The predominance of White and cisgender service providers was less welcoming to youth of marginalized identities. Several community stakeholders in Toronto and Montreal suggested that White people were “not sensitive to the realities” of the populations they were serving (Table 2). A lack of culturally sensitive service provision often resulted in SIY, especially those who were newcomers, racialized, 2SLGBTQ + and/or Indigenous being in the position of “having to teach and educate their doctors” (HCP, Toronto; Table 2). Across all sites in Canada and Kenya, poor experiences within institutional spaces and with care providers contributed **to lack of trust and associated fear** (dominant barrier at the intrapersonal level) which consequently limited access to healthcare for SIY (Table 2).

**Restrictive service provision** presented as a significant barrier at the institutional level. Participants across sites expressed the lack of primary care physicians that specialize in care for diverse SIY populations and travelling long distances and enduring long wait times to see those who did, contributed to heightened feelings of anxiety for SIY (Table 2). Service hours for healthcare were reported as restricted or at times that SIY considered inconvenient for SIY (e.g., 9am-5pm, Monday to Friday with no evenings or weekends) and associated fines with arriving late or not showing up discouraged SIY from making return appointments (Table 2). Specialized services for HIV care were described as particularly challenging to access due to limited appointments and strict clinic hours. Healthcare providers and community stakeholders noted how the “physician who specializes in HIV has a clinic from 9 − 4 on the first Thursday of the month” and that “the mobile clinic is once every two weeks for three hours… that’s not enough.”

Providing care in healthcare silos was another form of restrictive service provision. As suggested by one SIY, “more organizations need to collaborate”. This was especially important for SIY who required care from multiple care providers to address their needs relating to hormone therapy, mental health and HIV. This was also important for those recently diagnosed with HIV. There were reports of limited post-test counselling and newly diagnosed SIY being released back on the street with limited understanding of their diagnoses with no plan for follow-up care. To avoid this, sometimes HIV test results were withheld from youth.

“You might end up getting positive results but because this child has no owner or support the doctor might decide to keep quiet to avoid trauma and stress to this child…in the end we do not have a specific place where a street boy might not be counseled or treated” (CS, Kitale)

Across all sites healthcare providers, community stakeholders, and SIY also expressed how **inadequate HIV educational outreach** presented as a dominant barrier for accessing appropriate levels of care. In Kitale, youth were described as being “illiterate”, “not having the knowledge”, with only a “small number knowing that they should [go] for testing”. Healthcare providers noted how the services at the clinic were available but that they were under-utilized.

#### Interpersonal

**Ineffective communication from service providers** was an important barrier for SIY trying to access HIV-related care. Doctors were described as using challenging vocabulary that was hard for youth to understand and SIY felt their health concerns and opinions were not taken seriously (Table 2). Other youth felt healthcare providers did not communicate the appropriate information or the information that the SIY was seeking. For example, after receiving a positive HIV test one SIY described their healthcare provider gave them limited information on what they should do next. Doctors were described as “withholding information from [them] and not telling [them] everything” (SIY, Toronto). Some healthcare providers agreed with this characterization of healthcare provision and suggested a lot of these actions were rooted in stigma.

Some SIY felt healthcare providers questions to be too many and invasive. Without a trusting relationship, SIY were unwilling to share information about their sex life, their partners, their gender-affirming transition, and their sexuality. One SIY described her friend: “she hates going simply because the doctors ask too many questions about her sex life…they make it seem like something’s wrong with having sex, like you shouldn’t be having sex” (Toronto). When the answers to the doctors’ questions were not delivered, SIY described instances where doctors would not provide HIV testing.

“I’ve had a lot of crappy experiences where people won’t test me which I think is ridiculous” (SIY, Montreal)

#### Intrapersonal

Most youth were described as having a **low perception of need for healthcare**. Primarily across the Canadian sites, CS’s and HCP’s shared how the youth they worked with made comments such as, “[HIV] won’t happen to me”, “I look healthy” and “I feel healthy.” These sentiments suggest that SIY have an inaccurate understanding of HIV and exposure risks.

Healthcare providers and community stakeholders also described **lack of self-esteem** and poor self-validation as barriers for SIY. In Eldoret, community stakeholders found that SIY did not “value themselves, acted reckless” and internalized a lot of the societal-level stigma that they encountered while living on the streets. In the face of abject poverty, lack of basic needs, and insufficient social support, some SIY were viewed as lacking motivation for self-care (Table 2). A sense of hopelessness was pervasive when accessing HIV services was described as useless because “[They’re] going to die anyways,”(SIY, London).

“There’s a lot of self-judgment from youth for failing at things, what they consider failing but in reality the system was failing them” (CS, Montreal)

**High risk behaviours** such as unprotected sex, sharing needles, and excessive substance use were also commonly reported among SIY. One healthcare provider noted that “if a [SIY] is worried about getting a STI [and] housing, they’re going to choose housing and will engage in risky sex so that [they] can get a house for a night” (Toronto). Another healthcare provider suggested that the “majority of [SIY] are drug abusers from a very tender age” (Kitale) and that this behaviour resulted from a lack of basic needs. Participants also shared how comorbidities, such as poor mental health, acted as a driver for SIY to engage in these behaviours (Table 2). Adherence to HIV medication can also become limited because, as suggested by CSs and HCPs, “heavy substance may cause [SIY] to forget where they put their medication” (London) and can allow SIY to “confuse HIV drugs with other ones that they are taking” (Kitale).

### Facilitators to HIV service access

#### Societal

No facilitators were identified at the societal level by study participants.

#### Public policy

**Affordable HIV services and treatment** were major facilitators for SIY to access HIV care. Although limited, select government benefit programs made many HIV services free or relatively affordable in Canada and Kenya. Healthcare providers and community stakeholders expressed how “providing [SIY] with free services, testing, linkages, and other support” (Kitale) as well as “free antiretroviral medication like PrEP and PEP” (Montreal) increased access to HIV care among this population. In Canada, provincial benefit programs such as the Ontario Disability Support Program (ODSP) and Ontario Health Insurance Plan Plus (OHIP+) provided youth the opportunity to receive free HIV treatment after being diagnosed and individuals working at the community level often connected SIY to these resources.

“I can say to people, ‘You can get care… you can live a limitless life with HIV’, but then if I say, “Okay, you have to buy drugs to do that,” then that’s a huge barrier. But now with OHIP-plus, now I can say, ‘Okay, it’s yours for free’ (HCP, London)

#### Institutional

**Available and accessible HIV prevention tools** and **HIV awareness and education** were two dominant facilitators across all sites. In term of tools, harm reduction supplies, such as condoms, new needles, and PrEP, were known as important mechanisms for decreasing HIV risk and HCP’s and CS’s made efforts to make these available and accessible in sites where SIY frequently visited (Table 3). For HIV awareness and education, some inclusive practices included institutional commitments to hire more racially diverse and/or 2SLGBTQ + clinicians and staff. Some HCP’s and CS’s also noted the importance of having culturally safe material as this “really helps folks access healthcare with an HIV perspective”. Some community-based programs incorporated programs specifically for Indigenous and Two-Spirit youth to do drumming, smudging, and land acknowledgment at their events. Others used religion and spirituality to talk to SIY about the challenges they are facing (Table 3). Targeted education on harm reduction and HIV medication adherence were viewed as important strategies to increase healthcare uptake and decrease health risks. Communication and education tools that were adapted for transgender populations facilitated service access.

“Once we changed our communication tools, we had more trans people coming to see us, because they felt like we called out to them more.” (CS, Montreal)

Several healthcare providers and community stakeholders recognized that those who are at increased risk of or are living with HIV also face challenges with other health conditions, housing, and basic needs, including food and clothing. Therefore, **holistic models of care** were important facilitators to increasing access to services. Where available, care providers assisted SIY with their finances through government programs, helped them find stable housing, and other social supports. Holistic HIV services included mental healthcare, access for food, and accompaniment to appointments so that difficult medical jargon and the complex service systems could be explained in youth-accessible language. In Kitale, SIY who were afraid to access care due to lack of money were aided by social workers who found ways to “waive their bills and figure it out for them”.

#### Interpersonal

**Systems navigation support** from service providers was a notable mechanism that facilitated youth’s testing and treatment as was **peer support**. Building trusting relationships with care providers resulted in SIY attending more regular care visits. Healthcare providers described supportive clinician as being able to create spaces where SIY can ask questions “instead of receiving wrong answers from other sources”. One healthcare provider described an open-door policy: “if you can’t get an appointment one afternoon, that’s okay, show up, [I] will see you, don’t leave”. Following a person-centred approach was favorable and included “not being in a rush”, “making time for people”, and having the “right attitude” towards SIY. The holistic approach fostered SIY to feel understood and comfortable when receiving care.

Peers were described as a source of all-encompassing care that included accompaniment to appointments, helping them find shelters, as well as emotional support and companionship. Peers had shared lived experiences that “bridged gaps” with the medical care system by “taking youth through the various processes” and “alleviating tensions” for SIY who may have had previous negative experiences. SIY valued peers with diverse and multiple social identities. Racialized and 2SLGBTQ + youth preferred and trusted peers who shared their experiences and culture. They felt the healthcare system had a predominance of White cisgender men who were “trying to be a saviour” (CS, Montreal). Being of similar or only slightly older age also played an important role in establishing trust with peers. Whereas younger service providers were deemed less likely to be discriminatory.

“I’m a recovering addict and the people who have brought me the most support have been people going through the same thing as me, be it sex workers, people who have been to prison or are seropositive, it was always helpful for me. They understood me, they were in the same spot. I believe it’s essential to have access to peer support” (CS, Montreal)

Peers living with HIV were also described as helpful to SIY as those “who are receiving the service see themselves in the young person that is providing the support” (CS, Toronto). SIY noted learning about HIV, AIDS, and PrEP though their peers. Peers also encouraged youth to access harm reduction and accompanied them to receive HIV care. In particular, SIY were motivated to pursue taking care of their health recognizing that their peers with HIV are healthier (Table 3).

In addition, **personal relationships** motivated SIY to know their HIV status, to use ART, and to practice HIV prevention so that the people they cared fore could avoid infection. Intimate partner relationships specifically, acted as a support when youth felt afraid to get tested and learn of their diagnosis (Table 3).

#### Intrapersonal

A notable facilitator to accessing care was the **self-efficacy** of SIY themselves. Despite their circumstances, SIY’s had capacity and abilities to carry out necessary actions to prevent, test, and treat HIV. Across sites, SIY were described as having a “willingness to change their lifestyle” and “motivation to accept and adhere [to treatment]”. Others were described as taking care of their health because they wanted “to finish school”, “get a job”, and “acquire a stable home”. One healthcare provider described how youth “wanted to take care of [themselves], be healthy, and ensure that [they’re] not going to harm anyone” (London). Some SIY also self-advocated so that care providers understood their needs.

“Like ‘please don’t call me by that name. Please use my preferred name’... ‘This is all confidential’ making sure the places they go to are accessing respect that…‘I only use this card because legally I have no choice at this point’. And, you know, ‘I’m a citizen, I’m entitled to this’” (HCP, Toronto)

SIY were motivated to secure a stable future for themselves and sought safety for others who were struggling. Healthcare providers noted how youth often pursued treatment “to ensure [they] wouldn’t harm anyone” (London). Those engaged in commercial sex work were described as being “on top of their sexual health” and diligent about their routine check-ups. Learning how to cope with their own challenges motivated one SIY to want to be a social worker “so that [they] can help others too” (Toronto).

## Discussion

This study identified numerous modifiable barriers to, and facilitators of, accessing HIV prevention, testing and treatment for SIY in Kenya and Canada (Fig. 1). The barriers identified, though varied, were quite similar across all sites. For example, some of the most significant themes across the sites were intersectional stigma and discrimination at the societal level and inadequate support for basic needs at the policy level. Challenges at the institutional and interpersonal levels consistently reported a lack of understanding of the complexities of SIY’s lives and poor communication from service providers. Being intimately shaped by barriers on higher levels of the SEM, lack of trust and associated fear, perception of being healthy and having no need for healthcare, as well as poor self-esteem, limited HIV services utilization among SIY at an individual level. Conversely, multi-disciplinary and patient-centered HIV services were expressed as consistent facilitators, along with systems navigation and peer support which reportedly increased access to HIV services. In addition to this, inherent self-efficacy among SIY played a foundational role. With very little known about the HIV needs of SIY in both high- and low-income settings, these findings provide a strong foundation to bridging the gaps for this population and reaching the targeted 95% individuals knowing their HIV status, 95% people living with HIV on treatment and 95% of people living with HIV being virally supressed by 2030 set by UNAIDS [24].

Many of the main themes found were interrelated and – like a domino effect – factors on the societal and policy levels influenced and, in some cases, created barriers and facilitators on the institutional, interpersonal, and intrapersonal levels. Intersectional stigma and discrimination, due to complex adversities related to social inequality and oppressive identities, were often experienced by youth marginalized by poverty and multiple converging identities [42, 43]. This includes characteristics such as race, gender identity, sexual orientation, homelessness, and health status. In this study, participants shared hesitancy in being tested for HIV because of societal perceptions about being HIV-positive while already facing stigma related to being a young person, street-involved, and/or belonging to a racial, sexual, and/or gender minority group. Numerous studies have highlighted similar impacts among populations who not only bear the burden of HIV stigma and discrimination but also experience racism, homophobia, and transphobia, as well as the stigma associated with being homeless.[44].

Intersectional stigma also had negative implications in healthcare settings at the institutional level where there was limited education and lack of adequate training for service providers on the needs of diverse populations. Various populations have previously been identified as needing culturally competent and culturally safe care. Marshal et al., found an urgent need for culturally appropriate HIV information for Indigenous youth living in Canada [45]. Logie et al. found that overlapping institutionalized stigma towards sex workers and transgender women made it difficult for them to find tailored health services [41]. Stigma and discrimination often resulted in poor verbal and non-verbal communication at the interpersonal level between individuals in all three stakeholder groups in Canada and Kenya. Experiences shaped by negative interactions with care providers may play a role in promoting fear and distrust among SIY [46]. Additionally, SIY internalized many of the negative experiences of stigma and discrimination they were subjected to throughout their lives and this was often expressed at the individual level as reduced self-worth and engaging in HIV risk behaviours. These findings are consistent with the experiences of racialized transgender individuals living in Canada who had increased engagement in HIV related high-risk behaviours following experiences of racism and transphobia [47]. Reports of engagement with survival sex and injection drug use among SIY are interpreted with sensitivity to reflect how system level factors, such as social and institutional stigma, make it difficult for SIY to engage in beneficial or safe coping strategies.

In parallel to the barriers, identified facilitators at the policy and institutional levels often reinforced those at the interpersonal and intrapersonal levels. Holistic care concepts supported SIY in various components of their life instead of focusing solely on the biological factors related to HIV. In Canada and Kenya, various stakeholders connected SIY to housing, financial, and social support networks and took the steps necessary to navigate complex institutional structures with them. Coupled with affordable services and availability of evidence-based HIV tools such as PrEP, relationships that were built on trust and understanding provided SIY with several long-term benefits that increased their access to care. Interpersonal relationships with healthcare provider, community stakeholders, family, and peers have been cited as being just as, or even more, important than prevention programs that provide condoms or needles [48]. In LMIC, peer support programs are a cost-effective solution that have shown promise in increasing knowledge about and use of HIV services [31, 49]. Through example, peers in our study were reported to be able not only a strong pillar of support but could also motivate SIY to recognize their potential, effectively navigate health systems, take charge of their own care, and also protect others from harm.

There are three notable strengths to this study. First, there is minimal research regarding the experiences of SIY accessing HIV prevention, testing, and treatment in low-income settings and in specifically marginalized populations in high-income settings and this study helps to fill that gap. Second, this research highlights the perspectives of diverse stakeholders in distinct contexts, thus rendering our findings more generalizable. Additionally, three distinct formats of data collection including key informant interviews, FGD, and theatre testing, were used to understand these various perspectives.

This study also has limitations. The use of qualitative methods presents potential selection and response bias. It is hoped that the use of multiple qualitative methods enabled participants to respond freely in a format most comfortable for them. Participants were not asked for demographic information besides the location and type of respondent they were. The lack of information regarding participants’ race and ethnicity, gender identity, and sexual orientation limited our ability to ‘dive down’ into the impacts of specific identities on our findings. As part of the larger PNP study, information regarding the PN intervention was shared with participants and this may have influenced their decision to speak of the benefits of peers and systems navigation. Fourthly, participants were not asked to identify barriers and facilitators according to the SEM. Consequently, complex and often interrelated factors shared by SIY, healthcare provider, and community stakeholders were interpreted and assigned to the various levels of the SEM based on the perspectives of the authors. Finally, we were limited in our ability to engage healthcare providers, due to challenges with recruitment, likely resulting from their schedules and priorities in providing patient care rather than participating in research.

## Conclusion and recommendations

Through this study, we identified several common and often modifiable barriers and facilitators for SIY in accessing HIV services. Some issues created downstream barriers at the institutional, interpersonal, and intrapersonal levels of the SEM. Intersectional stigma and discrimination played a significant role in restricting SIY from accessing resources and played a dominant role in poor communication from service providers. Training and sensitization for healthcare providers about the intersectional issues facing street-involving youth in accessing HIV services could be critical to overcoming this barrier. [46] Provision of national health coverage to this population would also remove a key structural barrier arising as a result of extreme poverty. This study suggests that peer-based support services, such as peer navigators who share lived experiences with SIY, is one potential strategy for overcoming some of these challenges and accessing populations that are often hard to reach.

Additional facilitators, notably multi-disciplinary and patient-centered care has the potential to overcome barriers by being able to establish long-term relationships and increase self-efficacy among SIY. Although novel technologies to address HIV are evolving, there is a pressing need to facilitate access to existing care services and technologies to enable this population to make use of them.

## Electronic supplementary material

Below is the link to the electronic supplementary material.

Supplementary Material 1

## Data Availability

The datasets generated and analyzed during the current study are not publicly available due to limits of regulatory approvals but are available from the corresponding author on reasonable request. Codes used in the analysis of the qualitative data are also available upon request.

## Abbreviations

FGD: Focus group discussions
SEM: Socio-ecological model
SIY: Street Involved Youth
2SLGBTQ+: Two-Spirit, Lesbian, gay, bisexual, transgender, queer, and questioning
