# Supplementary material for: Barriers to and facilitators of accessing HIV services for street-involved youth in Canada and Kenya
Source: BMC Public Health. 2022 Oct 12;22:1901. doi: 10.1186/s12889-022-14290-7 (PMC9555255; doi:10.1186/s12889-022-14290-7)
Supplement: Supplementary file 1 — Supplementary Material 1 [file 12889_2022_14290_MOESM1_ESM.docx]

**Appendix A**

**Table A1. Supplementary quotes for barriers in Canada and Kenya**

| **Level** | **Theme** | **Illustrative Quotes** |
| --- | --- | --- |
| Societal Level | Intersectional Stigma and Discrimination | *“*Most Indigenous people don’t have a good experience with doctors, nurses, and dentists and so we avoid them at all costs until there’s an emergence.” (CS, Toronto) |
| Public Policy Level | Inadequate Access to Basic Needs | “If you live under the bridge, you don’t give a hoot about HIV meds. And why would you? If you haven’t eaten today, why would you care about taking a pill? So that has to be done first always.” (HCP, London) |
|  | Limited Community-Based Funding | “We had to give the same workshop to mainly heterosexual grade 7 students that we did when we went to an LGBTQ drop-in for youth who were in their 20s… I feel like something that would have been better for that one group is if that agency had the money, hiring someone from that community to speak about the subject, maybe talking about their own lived experience and talking about things that they’ve learned along the way from their community and sharing it back with the community.” (CS, Toronto) |
|  | Legal Documentation | “They need to have an address… to have a phone number...and those are legal requirements that they ask, there’s no leeway in that, which makes it really hard because we want to be there for the youth but the system itself doesn’t make it so we can.” (CS, Montreal) |
|  |  | “It’s always a problem, because the name on it is the one I need to take down. It can make people uncomfortable in the waiting room, the way they’re being welcomed. That’s an obstacle.”  (CS, Montreal) |
|  | Lack of Health Insurance | No additional quotes to report on this level |
| Institutional Level | Lack of Diversity and Inclusive Education and Training | “Lack of family doctors who are informed of [SIY] culture and the language that’s used when advertising the testing, the kinds of questions that are asked and screened for, the ways in which follow up is offered, like understanding of sex and intimacy in many ways are so culturally specific, and they’re not.” (HCP, Montreal) |
|  |  | “When I did my nursing program– the program itself was extremely transphobic. Like it was very much gender in two minorities, like women and male and everything gets described as that…there’s no training about trans folks. I had to train my classmates on it when I was in school. And the way they taught us about race was horrible, like it was extremely racist, condescending, and very problematic.” (CS, Montreal) |
|  |  | “I don’t feel like there’s any transgender people working in hospitals.” (SIY, Toronto) |
|  | Restrictive Service Provision | “There’s just a lot of waiting....you wait to see the doctor, you wait for your results, I don’t like a lot of waiting makes me anxious” (SIY, Toronto) |
|  |  | “If you have to jump through hoops, I find youth don’t end up or are less likely to make it there” (CS, Toronto) |
|  |  | “Like I find expecting someone to show up to an appointment that you make three weeks ago when someone doesn’t even have a way to write down that information…it is extremely hard not having a phone…” (CS, Montreal) |
|  |  | Some hospitals and clinics in Kenya were described as having security personnel who “had been warned not to allow any street children from getting treatment.” (CS, Eldoret) |
|  | Inadequate HIV Education Outreach | “Majority of the youth didn’t think that HIV was a thing anymore. ..They were just confused…Like ‘isn’t that a thing from the ‘80s? HIV is still around?’” (CS, Toronto) |
| Interpersonal Level | Ineffective Communication from Service Providers | “It’d be nice to just get more people who actually are going to treat me seriously.” (SIY, London) |
| Intrapersonal Level | Lack of Trust and Associated Fear | “I think for trans youth especially it can be scary to go see any sort of professional because they might have either experienced transphobia or they might have heard about people experiencing transphobia with doctors. Especially for sexual health testing and things because if they’re not really sure what that involves, they might be worried that they have to strip and all of this stuff and talk about their sex lives which can be kind of intimidating. And then usually there’s one or two service providers in any of those situations that will say something sort of off-putting, so that can be discouraging for people.” (CS, London) |
|  |  | “Fear of partners, parents, and the social network finding out when for example, a healthcare agency leaves a voicemail on the only means of contact.” (CS, London) |
|  |  | “Healthcare in general is… it’s very anxious thing for me.” (SIY, Toronto) |
|  | Low Perception of Need for Healthcare | No additional quotes to report on this level |
|  | Lack of Self-Esteem | “It’s very hard when they’ve been told you don’t matter…you shouldn’t exist… you’re garbage” (CS, London) |
|  |  | “There is this point of denial and this might be as a result of what this client has gone through. He sees no benefit of life and therefore it makes him or her to just go by the winds they do not care about themselves” (CS, Kitale) |
|  |  | “A lot of times they feel like they’re bothering us when we help them” (HCP, London) |
|  | High risk behaviours | “Sometimes it’s not a choice… people have many different reasons on why they use. Personally, I use to cover my pain. It’s the only way that I have found that I could cope with it. It’s by self-medicating and if I don’t, I find myself rather unhappy and just not comfortable living my day-to-day life” (SIY, Toronto) |
|  |  | “When I were using [intravenous] drugs, sharing needles, and having a lot of unprotected sex I was high-risk and it was so much harder for me to get treatment and care but now that I low risk it’s a lot easier.” (SIY, Montreal) |

**Table A2. Supplementary quotes for facilitators in Canada and Kenya**

| **Level** | **Theme** | **Illustrative Quotes** |
| --- | --- | --- |
| Societal Level | No facilitators were identified on this level by study participants | |
| Public Policy Level | No additional quotes to report on this level | |
| Institutional | Available and Accessible HIV Prevention Tools | “I think needles are actually less of an issue than they used to be with how readily available harm reduction supplies are.” (CS, Toronto) |
|  | HIV Awareness and Education | “We have made them know the importance of taking this drugs. We have also made them realize that if you are HIV positive and you start taking drug then you will have to take them for the rest of your life. They know that when they miss those drugs they can either fall sick or get infected.” (CS, Eldoret) |
|  |  | “We talk about the Bible, we teach the scripture, we teach them about God’s plan for family, we talk about issues that have caused them to run away from home.” (CS, Kitale) |
|  | Holistic Models of Care | “Our program, slowly shifted to be about challenging people’s social ideas about HIV, about testing, about sexuality and less about the medical knowledge.” (CS, Toronto) |
| Interpersonal Level | Systems Navigation Support | “It’s definitely good to have somebody not holding their hand but walking with them.” (CS, London) |
|  |  | “I guess the biggest traction we’ve gained as a community is youth really refuse to care about themselves unless they see that other people care about them first…the youth are invested because they know they have a person who cares about them, not just who’s paid to take care of them.” (CS, London) |
|  | Peer Support | “Even just seeing that a peer has done it and has succeeded.” (HCP, London) |
|  | Personal Relationships | “My partner he needs me just as much as I need him and I’m no help to him if I’m sick” (SIY, London) |
